# Spontaneous Epiretinal Membrane Resolution and Angiotensin Receptor Blockers: Case Observation, Literature Review and Perspectives

**DOI:** 10.3390/biomedicines11071976

**Published:** 2023-07-12

**Authors:** Filippo Confalonieri, Xhevat Lumi, Goran Petrovski

**Affiliations:** 1 Department of Ophthalmology, Oslo University Hospital, Kirkeveien 166, 0450 Oslo, Norway; filippo.confalonieri01@gmail.com (F.C.); xhlumi@hotmail.com (X.L.); 2 Center for Eye Research and Innovative Diagnostics, Department of Ophthalmology, Institute for Clinical Medicine, University of Oslo, Kirkeveien 166, 0450 Oslo, Norway; 3 Eye Hospital, University Medical Centre Ljubljana, Zaloška Cesta 2, 1000 Ljubljana, Slovenia; 4 Faculty of Medicine, University of Ljubljana, 1000 Ljubljana, Slovenia; 5 Department of Ophthalmology, University of Split School of Medicine and University Hospital Centre, 21000 Split, Croatia

**Keywords:** epiretinal membrane, fibrosis, angiotensin receptor blocker (ARB), angiotensin-converting enzyme inhibitor (ACE-I), vitreoretinal surgery

## Abstract

Introduction: Epiretinal membrane (ERM) is a relatively common condition affecting the macula. When symptoms become apparent and compromise a patient’s quality of vision, the only therapeutic approach available today is surgery with a vitrectomy and peeling of the ERM. Angiotensin receptor blockers (ARBs) and angiotensin-converting enzyme inhibitors (ACE-Is) reduce the effect of angiotensin II, limit the amount of fibrosis, and demonstrate consequences on fibrinogenesis in the human body. Case Description and Materials and Methods: A rare case of spontaneous ERM resolution with concomitant administration of ARB is reported. The patient was set on ARB treatment for migraines and arterial hypertension, and a posterior vitreous detachment was already present at the first diagnosis of ERM. The scientific literature addressing the systemic relationship between ARB, ACE-Is, and fibrosis in the past 25 years was searched in the PubMed, Medline, and EMBASE databases. Results: In total, 38 and 16 original articles have been selected for ARBs and ACE-Is, respectively, in regard to fibrosis modulation. Conclusion: ARBs and ACE-Is might have antifibrotic activity on ERM formation and resolution. Further clinical studies are necessary to explore this phenomenon.

## 1. Introduction

Fibrosis in different organs of the human body represents a growth, stiffening, and/or scarring of tissues, and it is characterized by excess deposition of extracellular matrix (ECM) components including collagen [1]. Fibrosis is also involved in the development of epiretinal membranes (ERMs), which consist of fibrocellular proliferation over the internal limiting membrane (ILM) [2].

The ERMs are among the most prevalent vitreoretinal diseases in all ethnicities [3,4,5,6,7,8,9,10,11,12]. They can be etiologically classified as primary (or idiopathic) and secondary, with the former being the most prevalent. While the pathophysiology of idiopathic ERM is not fully known [13,14], secondary ERMs can be seen in trauma, intraocular surgery, post-macular lasers, diabetic retinopathy, retinal vein occlusion, chronic macular edema, chronic intraocular inflammation, retinal detachment, and intraocular tumors [12,15].

Angiotensin receptor blockers (ARBs) and angiotensin-converting enzyme inhibitors (ACE-Is), beyond their function as antihypertensive drugs [16,17], are known to reduce scar formation through modulation of the angiotensin and TGF-β1 pathways in the fibrotic tissue [18,19,20]. Their role in ERM formation has not yet been explored.

Hereby, we report a rare case observation of spontaneous ERM resolution associated with the commencement of ACE-I treatment with a review of the literature on ACE-I and systemic fibrosis modulation to finally delineate future perspectives.

## 2. Case Description and Materials and Methods

**Figure 1:**
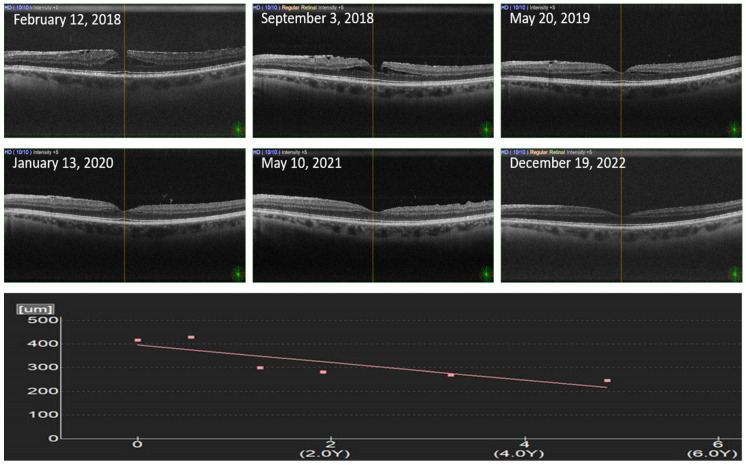
OCT progression of the ERM foveoschisis over the observation period, where the relief of the traction continued up to the last OCT scan. The central foveal thickness is shown below the OCT images over the observation years (OY).

A 58-year-old woman was referred to the Department of Ophthalmology, Oslo University Hospital, Norway, for a surgical evaluation of ERM producing metamorphopsia and perceived vision loss in the left eye in February 2018. The ophthalmic history revealed posterior vitreous detachment (PVD) 2–3 years before when she had received laser barrage treatment for a peripheral retinal tear at the 4 o’clock region of the left eye performed at a local eye clinic. The patient had been myopic since adolescence. Her best corrected visual acuity (BCVA) at the first observation was −0.2 logMAR with −4.00 sphere and −1.00 at 110° cylinder in the right eye, and −0.1 logMAR with −4.00 sphere and −0.75 at 70° cylinder in the left eye. At the slit-lamp examination, both eyes were within normality with clear lenses. The right eye showed a little atrophic peripapillary crescent compatible with moderate myopia and an inferotemporal area of pigment degeneration. In the left eye, fundoscopy also showed a little peripapillary atrophic crescent, an altered foveal reflex, and a peripheral laser barrage that had produced good retinochoroidal adhesion around the above-mentioned retinal tear. The first-observation OCT demonstrated ERM foveoschisis in the left eye (Figure 1). The subsequent follow-ups showed spontaneous resolution of the ERM that started between the first and the second observation, and continued up to the last eye examination (6 observations) over a period of 4 years, 9 months, and 1 week (from February 2018 to December 2022). No *pars plana* vitrectomy was indicated due to the good visual function and spontaneous resolution of the ERM. The BCVA did not change over the years of observation despite the drastic anatomical improvement.

Her systemic history showed obesity and incipient diabetes mellitus that resolved after a gastric bypass in 2015 followed by a 70 kg weight loss. Other systemic complaints were sleep apnea (treated with C-PAP) and migraines. At the first observation by the vitreoretinal surgeon, she was on dietary supplements, spray estrogen, metoprolol 50 mg qd for migraine attack prevention, and high blood pressure treatment. Since the arterial hypertension and migraine were incompletely controlled between the first and the second observation at the Department of Ophthalmology (February 2019 and May 2019), the patient was started on an ARB (Candesartan), which she continued to take thereafter at a dosage of 24 mg qd.

The scientific literature addressing the systemic relationship between ARBs, ACE-Is, and fibrosis in the past 25 years (since 1997) was searched in the PubMed, Medline, and EMBASE databases. Inclusion criteria were studies linking ARBs and ACE-Is to protection from fibrosis development in patients with systemic diseases. Exclusion criteria were review studies, pilot studies, case series, case reports, photo essays, and studies written in languages other than English.

## 3. Results

Fibrosis development in a wide spectrum of systemic conditions has been investigated in the past 25 years. None of these are related to the eyes or eye disorders. Thirty-eight original articles were selected for ARBs and fibrosis modulation, and sixteen for ACE-Is and fibrosis modulation. Table 1 and Table 2 summarize the studies included in the review for the ARBs and ACE-Is.

### 3.1. ARBs and Fibrosis

In the heart, ARBs have been shown to reduce the fibrogenic response in a myocardial-infarction-induced rat model [46], which has also been confirmed for Valsartan in another rat study [61]. Activation of the Ang AT(1) receptor was found to be an important factor in the development of pericardial thickening and collagen build-up in a pig model [62], the blockage of which could stop the development of pericardial fibrosis after heart surgery. In particular, the ARB (Candesartan) and another ACE-I (Temocapril) equally reduced ventricular fibrosis through different mechanisms in a hypertensive diastolic heart failure rat model [27]. Candesartan reduced the atrial fibrosis in a rat model through the suppression of connective tissue growth factor [63], while Losartan inhibited frizzled 8 and downregulated the WNT-5A pathway in an atrial fibrillation fibrosis reduction model in rats [42]. ARBs reduced fibrosis of the aortic valve in calcific aortic valve disease likely by lowering inflammation and interleukin 6 [25]. ARBs and neprilysin inhibitor (Valsartan and Sacubitril) prevented maladaptive cardiac fibrosis and dysfunction during pressure-overload-induced heart hypertrophy in a mouse model [64]. They also reduced fibrosis in isoproterenol-induced cardiac hypertrophy in a rat model [39]. ARBs (Valsartan) can improve cardiac fibrosis in diabetic nephropathy mice and achieve that by inhibiting miR-21 expression [40].

In the lungs, ARBs have been at least as effective as ACE−Is in reducing fibrosis development in radiation-induced lung fibrosis [29], both drug types being protective against radiation-induced pneumonitis and fibrosis by modulating TGF-β and alpha-actomyosin (αSMA) [65]. The use of an ARB (Olmesartan) demonstrated that both angiotensin 1 and 2 receptors are involved in fibrosis development in a mouse model of bleomycin-induced pulmonary fibrosis [43]. ARBs reduced lung fibrosis in a newborn rat model exposed to hyperoxia [66]. In particular, Losartan and calpain inhibition reduced pleural fibrosis in a mouse model [22]. ARBs and neprilysin inhibitors (Valsartan and Sacubitril) reduced fibrosis, pulmonary pressures, vascular remodeling, as well as right-ventricle hypertrophy in a rat model [67], while both ARBs and ACE-Is have been shown to possess a modulating effect in idiopathic pulmonary fibrosis [23].

ARBs have also shown efficacy in preventing radiation-induced fibrosis in the renal parenchyma of rats [68]. In a hypertensive rat model, a low dose of an ARB (Candesartan) reduced the fibroblast proliferation and TGF-β expression with a subsequent reduction in perivascular fibrosis [31]. An ARB (Losartan) reduced both the epithelial–mesenchymal transition and fibrosis development in a unilateral ureteral obstruction in a rat model [35]. This appeared to be active not only in unilateral ureteral obstruction but also in other renal diseases, therefore enhancing the beneficial effect of ARBs in kidney diseases. The same ARB was also effective in suppressing inflammation and fibrosis in the pancreas of a rat model, similar to what had already been demonstrated in the heart, kidney, and liver [69]. ARBs have been shown to improve the state of renal tubulointerstitial fibrosis [47]. In particular, Fimasartan has been shown to be effective in reducing renal oxidative stress, inflammation, and fibrosis in a unilateral ureteral obstruction mouse model [28].

An ARB (Candesartan) reduced liver fibrosis by suppressing collagen I and TGF-β1 expression as well as reducing hepatic stellate cell activation and the lipid peroxidation of proteins [56] through a therapeutic effect on cholestasis-induced liver fibrosis in rats. In another rat non-alcoholic steatohepatitis model, similar effects of ARBs were demonstrated in addition to a reduced production of aspartate aminotransferase [37,68]. The combination of ARBs and rifaximin achieved an additive affect against non-alcoholic-steatohepatitis-induced fibrosis in a rat model [30]. In a bile duct ligation rat model, the inhibitory effects of ARBs on hepatic fibrosis were found to be superior to those of ACE-Is [21]. Candesartan, at a regularly-used dose, was shown to be effective in reducing liver fibrosis in humans affected by chronic hepatitis C [48]. Short-term treatment with a hepatic-stellate-cell-selective drug carrier, mannose-6-phosphate-modified human serum albumin (losartan-M6PHSA), was also effective at reducing liver fibrosis [37]. An antifibrotic effect of an ARB (Telmisartan), which is an angiotensin 1 (AT) receptor blocker and a PPARγ partial agonist, was demonstrated in both acute and chronic stages of a Schistosoma-mansoni-induced liver fibrosis mouse model [38]. Hypertensive patients with non-alcoholic fatty liver disease receiving ARBs had less liver fibrosis than their counterparts not on ARB therapy [70]. Both Telmisartan and Losartan reduced inflammation and oxidative stress in a thioacetamide mouse model of liver fibrosis [24]. Ex vivo and in vivo, it has been demonstrated that ARBs (Losartan) reduce liver fibrosis in a mouse model [20].

In skeletal muscle injury in mice, ARBs were shown to reduce the fibrosis response, ultimately improving the healing process [51]. These drugs also reduced the fibrotic response in mice with normal and dystrophic skeletal muscles [36].

An ARB (Candesartan) significantly reduced TGF-β1 expression and suppressed tumor cell proliferation and stromal fibrosis in a mouse gastric tumor model [52].

Skin scarring in humans undergoing thyroid surgery had less fibrosis in patients on ARBs or ACE-Is [44].

### 3.2. ACE-Is and Fibrosis

As a pharmacological class, ACE-Is are a group of drugs that can reduce the availability of angiotensin II in the body. They are primarily utilized for the treatment of arterial hypertension, congestive heart failure, diabetic nephropathy, and many other cardiovascular conditions secondary to hypertension [16]. The influence of ACE-Is in the process of fibrosis has also been demonstrated in many studies.

In the heart, not only do ACE-Is inhibit the proliferation of cardiac fibroblasts at various levels, but they also hinder other mitogenic signals from estrogens [60]. The antifibrotic impact of ACE-Is on the heart is due to the suppression of N-acetyl-seryl-aspartyl-lysyl-proline (Ac-SDKP) hydrolysis, which results in a reduction of myocardial cell proliferation (most likely fibroblasts), inflammatory cell infiltration, TGF-β expression, Smad2 activation, and collagen production [58]. Transient ACE-I administration in hypertensive rats modulated cardiac fibroblast subpopulations and activation, resulting in reduced fibrosis and an overall reduced fibrogenic phenotype [53]. In particular, Captopril was able to reduce the scar area, fibroblast count, and capillary count in spontaneously hypertensive rats [71].

In the liver–bile duct–pancreas system, an ACE-I (Captopril) was shown to reduce TGF- β1 and collagen gene expression, delaying the progression of hepatic fibrosis in a rat model created by bile duct ligation [72]. Captopril was also able to suppress the hepatic stellate cell activation via the NF-kappaB or Wnt3α/β-catenin pathways, thus reducing fibrosis development in the liver [54]. In male WBN/Kob rats, ACE-Is (lisinopril) reduced the fibrosis characterizing chronic pancreatitis [41]. Specifically, Lisinopril inhibited TGF- β1 mRNA expression, preventing pancreatic stellate cell activation. In vitro, it was demonstrated that a combination of perindopril and interferon produces an antifibrosis effect on liver cells [55]. ACE-Is prevented the generation of proinflammatory cytokines in mouse models of colitis and colonic fibrosis, most likely through inhibiting the TGF-β signaling pathway, paving the way for an innovative inflammatory bowel disease treatment [34]. An ACE-I (Ramipril) was effective at reducing inflammation, oxidative stress, and fibrosis in carbon-tetrachloride-treated rat liver [45].

In the skin, the early administration of ACE-Is (Enalapril) reduced the fibrosis and scarring process on a dermal ear rabbit model [49], which was hypothesized to be driven by the downregulation of collagen production. This drug also inhibited the renal fibrosis induced by unilateral ureteral obstruction in rats, hypothesizing a mechanism driven by the inhibition of mast cell degranulation [57]. ACE-Is and ARBs (Ramipril and Losartan) reduced scar formation through hindering fibroblast proliferation, collagen, and TGF-β1 expression, and suppressed the phosphorylation of SMAD2/3 and TAK1, both in vitro and in vivo [18]. Similarly, ACE-Is have been shown to possess antifibrotic properties in scar formation in mice [26], affecting peptides that suppress the TGF-β1/Smad and TGF-β1/TAK1 pathways. The inhibition of both Smad- and TAK1-mediated pathways by ACE-Is could thus lead to new antifibrotic agents’ development.

ACE-Is reduced skeletal muscle fibrosis in the early phase after streptozotocin-induced diabetes in mice [59]. Ramipril reduced radiation-induced periprosthetic capsular fibrosis and contracture in breast surgery [50].

## 4. Discussion

We hereby show the implication of previous findings on the role of ARBs and ACE-Is in preventing fibrosis development in various organs of the body. The mechanism by which these drugs act upon pathways of ERM development, especially the TGF-β pathway, is being pointed out here [19].

**Figure 2:**
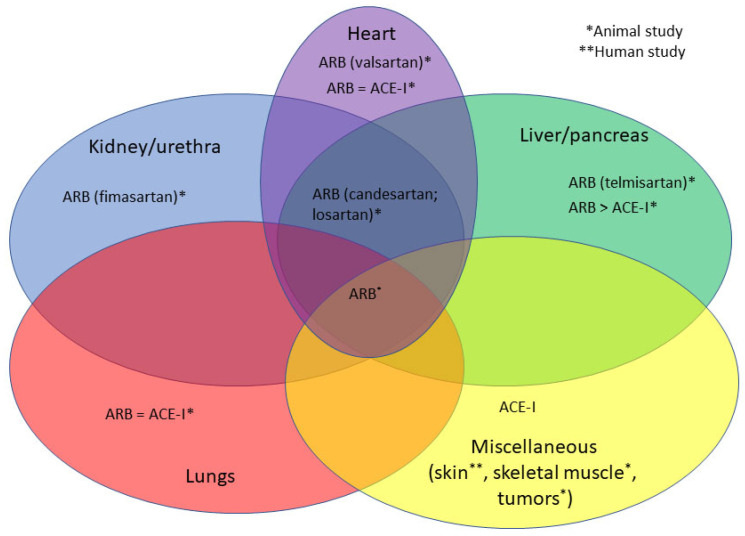
ARBs and ACE-Is used in fibrosis modulation in different organ systems and their overlaps.

Nothing is known about the role of ARBs and ACE-Is or TGF-β in ERM’s pathogenesis. Figure 2 summarizes the different overlapping ARBs and ACE-Is used in fibrosis modulation in different organ systems, showing ARBs (Candesartan and Losartan) to be the most ubiquitously used drugs affecting fibrosis.

ERMs are generated through a fibrosis mechanism involving various molecules, among which integrin β1, cathepsin B, epidermal growth factor receptor, protein-glutamine gamma-glutamyltransferase 2, prolow-density lipoprotein receptor-related protein 1, and TGF-β have been described [73]. In particular, TGF-β has been known to be a versatile cytokine that belongs to the TGF superfamily, and it is considered a major fibrosis modulator [74]. Furthermore, several pathways have been shown to be involved in the interaction between ECM, ECM-related molecules, cells, cell receptors, and intra- or extra-cellular proteins that can, in the end, contribute to the development of ERMs [73]. The process of ERM development is driven by more than 50 genes, among them being the Tumor Necrosis Factor (TNF), CCL2 (chemokine C-C motif ligand), Metastasis Associated Lung Adenocarcinoma Transcript 1 (MALAT1), TGF-β1, TGF-β2, Interleukin-6 (IL-6), IL-10, VEGF, and glial fibrillary acidic protein (GFAP) [75].

Since TGF-β is involved in other systems, particularly the immune system, direct targeting of TGF-β is unlikely to be therapeutically feasible [74].

**Figure 3:**
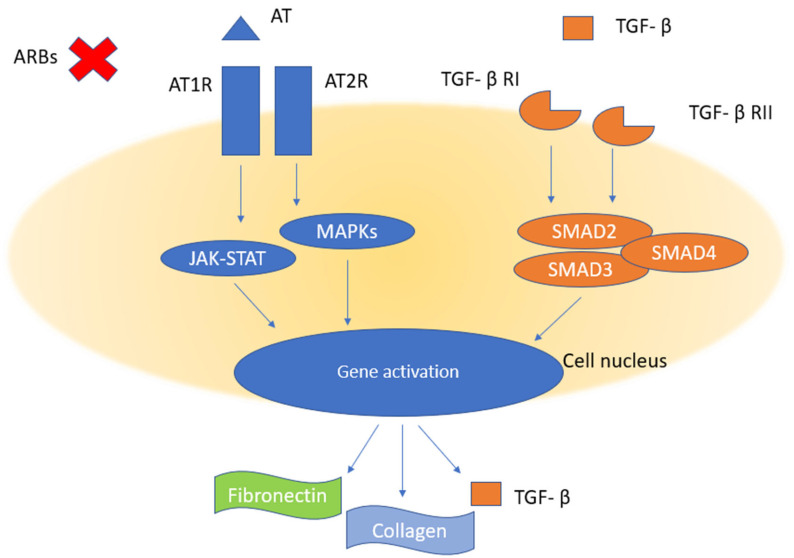
Proposed mechanism of action through which ARBs (and, similarly, ACE-Is) can reduce fibrosis development in ERMs and possibly bring about a resolution of the condition. Abbreviations: ARBs, angiotensin receptor blockers; AT, angiotensin; AT1R, angiotensin 1 receptor; AT2R, angiotensin 2 receptor; JAK-STAT, janus-kinase signal transducer and activator of transcription; MAPKs, mitogen-activated protein kinases; SMAD, mothers against decapentaplegic family transcription factors.

It has been previously reported that ERMs can spontaneously resolve in cases of PVD occurrence, and this may happen when the ERM’s adhesion to the posterior hyaloid membrane is stronger than its adhesion to the underlying ILM [76,77,78,79]. Since our patient was known to have an already complete PVD prior to the diagnosis of ERM and the intake of ARBs (candesartan) was the only evident discriminating factor that could have led to the spontaneous resolution of ERM, we hypothesize a molecular mechanism through which the fibrosis constituting the ERM could have been affected and resolved by the molecular mechanism of ARBs (Figure 3). In particular, we speculate that the TGF-β pathway could be the main molecular target among the different pro-inflammatory cytokine pathways that may be involved in the disease process, since it has been shown to be heavily inhibited by ARBs and ACE-Is [19]. These inhibitors, while acting upon the angiotensin receptor system (angiotensin 1 and 2 receptor (AT1R and AT2R respectively)), influence or reduce TGF-β expression and fibrosis in different organs of both animals and humans through modulating the JAK-STAT/MAPK intracellular pathways, which, in turn, influences or reduces the expression of fibronectin, collagen, and TGF-β itself [80,81,82,83].

## 5. Conclusions

To our knowledge, this is the first report showing a possible correlation between ARBs and ERM resolution, supported by clinical observations and a review of literature. In perspective, both ARBs and ACE-Is should be examined in further clinical studies to confirm their potential in the prevention and treatment of ERM.

## Figures and Tables

**Table 1:** Selected articles dealing with fibrosis modulation by ARBs.

Drugs Employed	Organs Involved
Candesartan [21,22,23,24,25,26] (and Temocapril [27])	Heart ^¥^, pancreas ^¥^, liver ^¥,^*, stomach tumor ^§^
Firmasartan [28]	Kidney ^§,§^
L-158809 (and captopril and enalapril and pioglitazone) [28,29,30]	Lung ^¥^, kidney ^¥^
Losartan [20,31,32,33,34,35]	Heart ^¥^, kidney ^¥^, liver ^§^, skeletal muscle ^§^, lung ^¥^
Losartan and irbesartan (and captopril and ramipril) [21]; (and calpain) [22]; (and rifaximin) [30]; (and obeticholic acid) [32]; (and valsartan) [23]; (or r ZD-7155) [36]	Liver ^¥^, pleura ^§^, skeletal muscle ^§^
Losartan-M6PHSA [37]	Liver ^¥^
Olmesartan [37,38]	Liver ^¥^, lung ^§^
Sacubitril and Valsartan [39,40]	Heart ^¥,§^
Telmisartan [38] (and Losartan [24])	Liver ^§,¥^, lung *
Valsartan [27,41,42,43]	Pericardium ^₤^, heart ^¥,§^
Various [44]	Skin *
N/A (ARBs and ACE-Is) [45,46]	Aortic valve * (post mortem), liver *

Model of study: rat ^¥^; mouse ^§^; human *; pig ^₤^.

**Table 2:** Selected articles dealing with fibrosis modulation by ACE-Is.

Drugs Employed	Organs Involved
Captopril [47,48,49,50]	Heart ^¥^, liver ^¥^, skin ^¥^
Enalapril [51,52,53,54] (and PEG)	Heart ^¥^, colon ^§^, kidney ^¥^, skin ^‡^
Interferon-β and Perindopril [55]	Liver *
Lisinopril [56,57]	Pancreas ^¥^, skeletal muscle ^§^
Ramipril [58,59] (and Losartan [18], hydralazine [26])	Liver ^¥^, skin ^¥^,^§^ capsular tissue around breast implant ^¥^
Moexiprilat [60]	Heart ^¥^

Model of study: rat ^¥^; mouse ^§^; rabbit ^‡^; human *.

## Data Availability

Data are available on reasonable request from the corresponding author.
